# Supplementary material for: Hyaluronidase-1-mediated glycocalyx impairment underlies endothelial abnormalities in polypoidal choroidal vasculopathy
Source: BMC Biol. 2022 Feb 15;20:47. doi: 10.1186/s12915-022-01244-z (PMC8845246; doi:10.1186/s12915-022-01244-z)

**Additional Information**

**Additional Figure S1**

**Additional Figure S1: Extrinsic mediators influence sprouting angiogenesis of BOECs.** BOECs were coated onto microcarrier beads and allowed to sprout either in standard culture media (EGM-2 with 10% heat-inactivated FBS) or media containing isogenic patient plasma (EGM-2 with 10% plasma) for 24 hours in a fibrin sprouting assay. Quantification of number of sprouts per bead, average sprout length and number of filopodia branch points per sprout was performed using Imaris; n = 10 beads per donor cell line. Data are presented as box and whiskers plots showing the median, minimum, maximum and quartiles of each group. *P* values between individual groups were obtained using two-way ANOVA with Tukey’s multiple comparisons test. ***p*<0.01, ****p*>0.001*.*

**Additional Figure S2**

**Additional Figure S2: scRNA-seq data quality checks and cluster marker enrichments. (a)**.Violin plots with dots representing individual cells for the number of features, number of counts, percentage of mitochondrial genes (mt) and percentage of ribosomal genes (rb) found in each individual library **(b)** Violin plots showing expression scores for S-phase and G2M genes in each individual library. **(c)** Heatmap showing expression of top 10 marker genes for each clusters at the single cell level. Dotplots of the top unique gene sets, GOBP in **(d)** and Reactome in **(e),** enriched for the upregulated marker genes of each identified cluster shown here ranked by Bonferroni corrected *p*-values. Right panel shows violin plots that represent calculated module scores for the processes indicated.

**Additional Figure S3**

**Additional Figure S3: Differential expression in PCV and normal BOECs after heterogeneous flow. (a)** Percentage breakdown of cells per cell state for individual PCV and normal libraries. **(b)** Gene expression profiling of cell state enriched genes in PCV and normal BOECs over prolonged exposure to heterogenous flow (6 hours, 1 day and 7 days). All graphs showing means with standard deviations (*n* = 3, **p*<0.05, ***p*<0.01, ****p*<0.001, *****p*<0.0001, two-way ANOVA with Sidak's multiple comparisons test comparing PCV and normal groups within timepoints). **(c)** Violin plots showing differential expression of ECM-modifying genes and other hyaluronidases detected in the study, *adj. *p*< 0.01.

**Additional Figure S4**

**Additional Figure S4: Differential expression in PCV and normal BOECs after heterogeneous flow. (a)** Western blot analyses of cell lysates from 4 PCV and 3 normal BOEC lines, subjected to 24h of heterogeneous flow, showing syndecan-2 (SDC2) detection at 23 kDa and Beta-actin as the loading control at 42 kDa. Right panel shows densitomeric quantification of SDC2 bands normalized against Beta-actin. Data are presented as mean with standard deviations. ns, non-significant (two-tailed t-test). **(b)** Fold change of *KLF2* gene expressions (normalized to static control) in 4 PCV and 3 normal BOEC lines after 6 hours of heterogeneous flow exposure, with heparan sulfate supplementation or vehicle control. All bar graphs showing means with standard deviations. *n* indicates number of cell lines evaluated for each group while *p*-value is from two-way ANOVA with Tukey’s multiple comparisons test, **p*<0.05.

**Additional Table S1:** Demographics details of PCV patients and normal individuals.

| **Disease status** | **Age** | **Blood outgrowth endothelial cells (BOECs)** | **Genotypes** | | |
| --- | --- | --- | --- | --- | --- |
|  |  |  | ARMS2  rs10490924  (risk allele: T) | HTRA1  rs11200638  (risk allele: A) | CFH  rs800292  (risk allele: G) |
| PCV | 71 | ✓ | TT | AA | GA |
|  | 86 | ✓ | TT | AA | AA |
|  | 73 |  | TT | AA | GA |
|  | 71 | ✓ | TT | AA | GG |
|  | 71 | ✓ | GT | GA | GA |
| Control | 46 | ✓ | GT | GA | GG |
|  | 46 | ✓ | GG | GG | GA |
|  | 71 | ✓ | GG | GG | GG |
|  | 49 | ✓ | GT | GA | GA |
|  | 47 | ✓ | GT | GA | GG |
|  | 46 | ✓ | GT | GA | GA |

**Additional Table S2**: HYAL1 validation in clinical samples.

| **Characteristics, N (%)** | **Plasma of PCV patients (n = 25)** | **Vitreous humor of PCV patients (n = 17)** |
| --- | --- | --- |
| Age* | 66.8 (11.5) | 69.9 (8.1) |
| Age, Male* | 67.3 (11.9) | 71.2 (9) |
| Age, Female* | 65.2 (11.3) | 67.7 (6.3) |
| Gender, male | 19 (76) | 6 (35.3) |
| Gender, female | 6 (24) | 11 (64.7) |
| Ethnicity | Chinese: 25 (100) | Chinese: 13 (76.4)  Malay: 2 (11.8)  Indian: 1 (5.9)  Filipino: 1 (5.9) |

All values are reported as N (%) where N indicted number of observations.

*Values are expressed as mean (± standard deviation).

**Additional Table S3:** Key resources used in this study.

| **REAGENT or RESOURCE** | **SOURCE** | **IDENTIFIER** |
| --- | --- | --- |
| **Antibodies** | | |
| Mouse monoclonal anti-CD31-APC (clone wm59) | BioLegend | Cat# 303116, RRID:AB_1877151 |
| Mouse monoclonal anti-CD144-PE (clone 55-7H1) | BD Pharmigen | Cat# 560410, RRID:AB_1645502 |
| Mouse monoclonal anti-CD45-APC (clone 2D1) | Invitrogen | Cat# 17-9459-42, RRID:AB_10718532 |
| Mouse monoclonal anti-CD68-FITC (clone Y1/82ª) | BioLegend | Cat# 333806, RRID:AB_1089054 |
| Mouse monoclonal anti-CD133-APC (clone 7) | BioLegend | Cat# 372806, RRID:AB_2632882 |
| Goat polyclonal anti-CDH5 | Santa Cruz | Cat# sc-6458; RRID: AB_2077955 |
| Rabbit polyclonal anti-VWF | Abcam | Cat# ab9378; RRID: AB_307223 |
| Rabbit polyclonal anti-SDC2 | Abcam | Cat# ab205884 |
| Rabbit polyclonal anti-HYAL1 | Thermo Fisher Scientific | PA5-79420, RRID:AB_2746536 |
| Rabbit polyclonal anti-beta-Actin | Abcam | Cat# ab75186, RRID:AB_1280759 |
| Mouse monoclonal anti-CAV1 | Thermo Fisher Scientific | Cat# MA3-600, RRID:AB_779568 |
| Goat polyclonal anti-CDH5 | Santa Cruz Biotechnology | Cat# sc-6458, RRID:AB_2077955 |
| **Primer sequences for qPCR** | | |
| **Genes** | **Forward primer** | **Reverse primer** |
| *GAPDH* | CCGTCAAGGCTGAGAACGG | CTCAGCGCCAGCATCGC |
| *CXCL8* | TTGGCAGCCTTCCTGATTTCTGCAG | ACAACCCTCTGCACCCAGTTTTC |
| *TAGLN* | ATGACAGGCTACGGACGAC | CAGGTGCAGTTACCATTGCTC |
| *TGFB2* | CAGCACACTCGATATGGACCA | GTTGTAGATGGAAATCACCTCCG |
| *ANKRD1* | AGAATGGCAATGGGGAGGCA | TCTTTTTGAGCTCTGCCTCTCGT |
| *CLDN5* | CTCTGCTGGTTCGCCAACAT | CAGCTCGTACTTCTGCGACA |
| *LYVE1* | CTGAAGGGGTAGGCACGAT | GACACCTGGATGGAAAGCTC |
| *AQP1* | TAACCCTGCTCGGTCCTTTG | AGTCGTAGATGAGTACAGCCAG |
| *CLU* | ACAAACGAAGAGCGCAAGACAC | GGTCTCATTTAGGGCATCCTCTTTC |
| *KLF2* | AGACCTACACCAAGAGTTCGCATC | CATGTGCCGTTTCATGTGCAGC |
| **Biological samples** | | |
| PBMC | This paper | N/A |
| Plasma | This paper | N/A |
| Vitreous humor | This paper | N/A |
| **Chemicals, peptides, and recombinant proteins** | | |
| Biotinylated-HABP (versican G1 domain) | Amsbio | AMS.HKD-BC41 |
| TRITC-Phalloidin | Merck | 90228 |
| MitoSOX Red | Thermo Fisher Scientific | M36008 |
| **Critical commercial assays** | | |
| Chromium Single Cell 3’ v3 Reagent Kit | 10X Genomics | PN-1000075 |
| Seahorse XF Cell Mito Stress Test Kit | Agilent Technologies | 103015-100 |
| HYAL1 DuoSet ELISA and DuoSet ELISA Ancillary Reagent Kit 2 | R&D Systems | DY7358 and DY008 |
| **Experimental models: Cell lines** | | |
| Patient-derived BOECs | This paper | N/A |
| **Oligonucleotides** | | |
| Non-targeting siRNA (ON-TARGETplus NT#4) | Dharmacon | D-001810-04-05 |
| siRNA pool for HYAL1 (ON-TARGETplus SMARTpool) | Dharmacon | L-010516-00-0005 |
| **Software and algorithms** | | |
| Seurat v 3.2.0 | Reference 74 in Main Manuscript | https://satijalab.org/seurat/ |
| clusterProfiler v 3.17.0 | Reference 77 in Main Manuscript | https://github.com/YuLab-SMU/clusterProfiler |
| ImageJ/Fiji | Reference 72 in Main Manuscript | https://imagej.net/Fiji/Downloads |
| ZEN BLUE | ZEISS | https://www.zeiss.com/microscopy/int/products/microscope-software/zen.html |
| FlowJo | Becton Dickinson | https://www.flowjo.com/ |
| Quest Graph™ Four Parameter Logistic (4PL) Curve Calculator | AAT Bioquest, Inc | https://www.aatbio.com/tools/four-parameter-logistic-4pl-curve-regression-online-calculator |
| Prism version 9.0.2. | GraphPad | https://www.graphpad.com/scientific-software/prism/ |
| Imaris 3.0 | Oxford Instruments | https://imaris.oxinst.com/ |
| **Others** | | |
| Synergy H1 | BioTek | N/A |
| Cytation 3 | BioTek | N/A |
| Confocal Airyscan Microscope LSM800 | ZEISS | N/A |
| CellDiscoverer7 | ZEISS | N/A |
| BD LSR Fortessa X-20 cell analyser | Becton Dickinson | N/A |

**Original Data**

**Uncropped scans of western blots for Figure 5a**

**
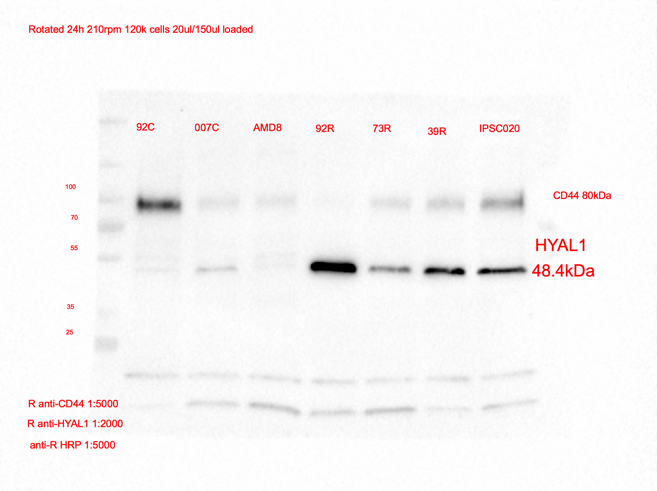

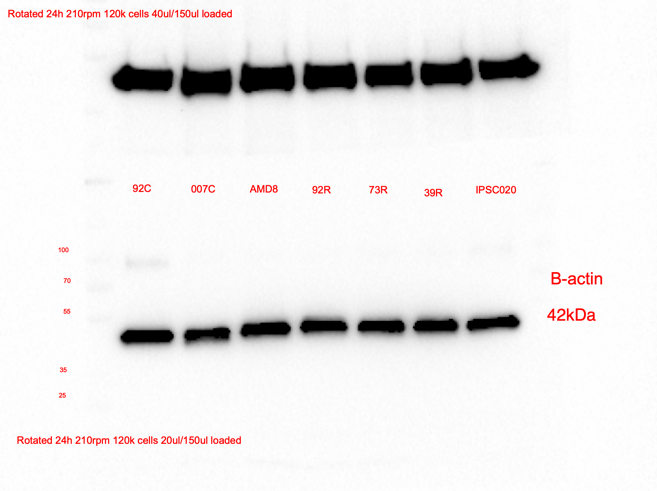
**

**Uncropped scans of western blots for Figure 6a**


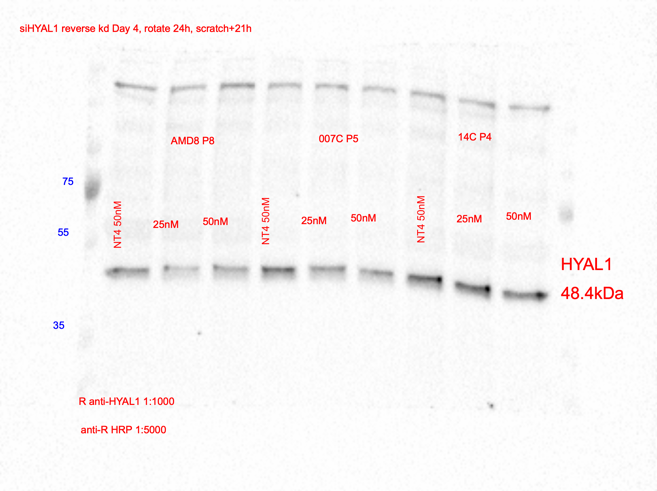

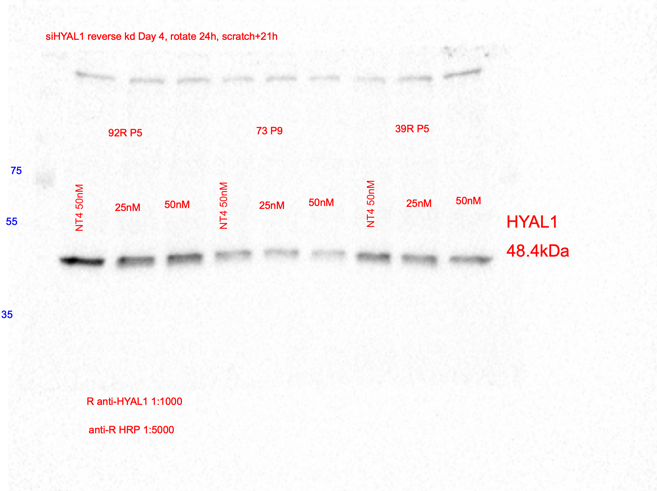

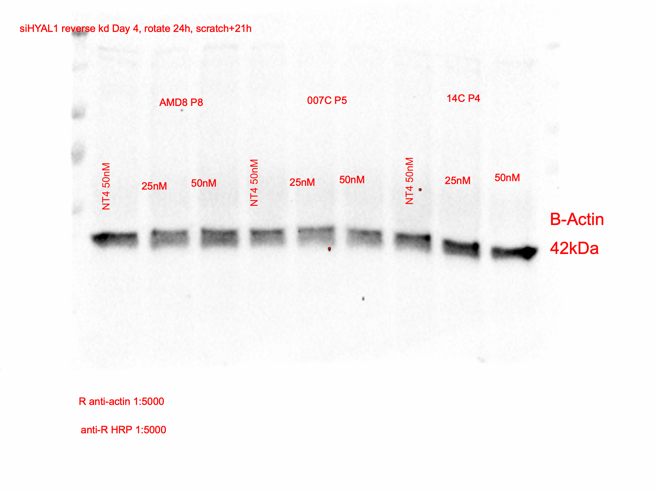

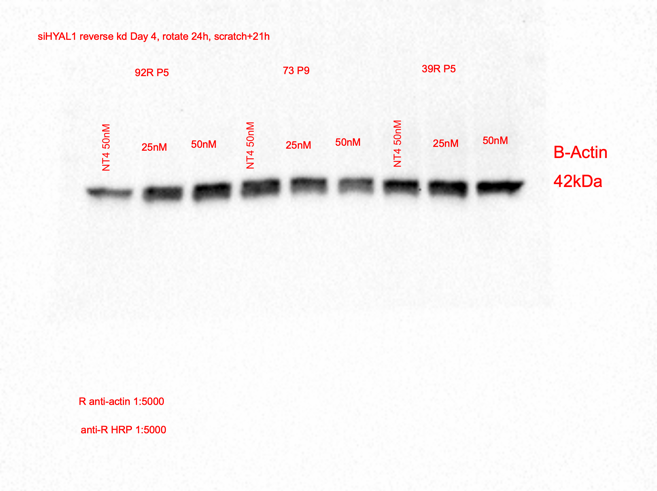

Supplement: Supplementary file 1 — Additional file 1: Figure S1. Extrinsic mediators influence sprouting angiogenesis of BOECs. Figure S2. scRNA-seq data quality checks and cluster marker enrichments. Figure S3. Differential expression in PCV and normal BOECs after heterogeneous flow. Figure S4. Differential expression in PCV and normal BOECs after heterogeneous flow. Table S1. Demographics details of PCV patients and normal individuals. Table S2. HYAL1 validation in clinical samples. Table S3. Key resources used in this study. [file 12915_2022_1244_MOESM1_ESM.docx]
